# Biological Characteristics and Fungicide Screening of *Bipolaris oryzae* Causing Leaf Spot on Banana in China

**DOI:** 10.3390/microorganisms13061285

**Published:** 2025-05-30

**Authors:** Yanxiang Qi, Hong Zhao, Zhaojing Zhang, Yanfei Ouyang, Xin Zhang

**Affiliations:** 1 Hainan Provincial Key Laboratory of Pests Detection and Control for Tropical Agriculture, Environment and Plant Protection Institute, Chinese Academy of Tropical Agricultural Sciences, Haikou 571101, China; zhaohong0430@163.com (H.Z.); 15092851239a@sina.com (Z.Z.); m18778970968@163.com (Y.O.); 2 State Key Laboratory of Green Pesticide, Key Laboratory of Green Pesticide and Agricultural Bioengineering, Ministry of Education, Center for R&D of Fine Chemicals, Guizhou University, Guiyang 550025, China; 3 College of Plant Science and Technology, Huazhong Agricultural University, Wuhan 430070, China

**Keywords:** banana leaf spot, *Bipolaris oryzae*, biological characteristics, antifungal activity

## Abstract

Foliar diseases caused by various fungi severely affect the yield and quality of banana crops. This study was conducted to clarify the biological characteristics of *Bipolaris oryzae* (teleomorph: *Cochliobolus miyabeanus*), a pathogen reported in 2023 as a new etiological agent of leaf spot in the banana variety ‘Pisang Mas’ (*Musa acuminata*, AA group) in Hainan Province, China, and to screen effective fungicides for its control. The results indicated that banana leaf extract agar (BLEA) and cornmeal agar (CMA) were the best media for the growth and sporulation of the pathogen, respectively. The pathogen grew best on a Czapek’s agar (CZA) medium with sucrose as a carbon source and yeast extract as a nitrogen source, while the optimal carbon and nitrogen sources for sporulation were lactose and beef extract, respectively. The pathogen could grow within a temperature range from 5 °C to 35 °C, and the optimal temperatures for growth and sporulation were 30 °C and 25 °C, respectively. Exposure to 50 °C for 10 min was lethal. Additionally, the pathogen could grow and sporulate within pH ranges of 4 to 10 and 4 to 9, respectively, and the optimal pH values for growth and sporulation were 5 and 8, respectively. The optimal photoperiods for growth and sporulation were 16 h light/8 h dark and 24 h light, respectively. Among the 12 tested fungicides, 500 g/L of iprodione SC showed the highest toxicity against *B. oryzae*, with an EC_50_ value of 0.08 μg/mL, followed by 30% difenoconazole-azoxystrobin SC and 125 g/L of epoxiconazole SC, with EC_50_ values of 0.13 μg·mL^−1^ and 0.20 μg/mL, respectively. A fungicide containing 40% chlorothalonil SC had the poorest fungicidal activity, with an EC_50_ value of 155.98 μg/mL. An artificial inoculation pot experiment showed that 125 g/L of epoxiconazole SC at 250 μg/mL, 500 g/L of iprodione SC at 1667 μg/mL, and 30% difenoconazole-azoxystrobin SC at 250 μg/mL provided a protective control efficacy of 100% against *B. oryzae*, while 125 g/L of epoxiconazole SC at 250 μg/mL and 500 g/L of iprodione SC at 1667 μg/mL provided a curative control efficacy of greater than 60%. This study clarified the optimal conditions for the mycelial growth and sporulation of *B. oryzae* isolated from banana and screened out fungicides with effective inhibitory activities. These results can provide guidance for field applications and the management of leaf spot caused by *B. oryzae* in banana.

## 1. Introduction

Banana (*Musa* spp.) originated in South-East Asia (the Indochina and Burma region), from which it spread across the tropical and sub-tropical regions of the world, and currently represents the fourth most economically important food crop after rice, wheat, and maize [1]. Banana is believed to have been brought to China 2000 years ago and has become an economically important fruit in the south of the country. At present, China is the second-largest producer and consumer of banana in the world [2]. In Hainan, banana is one of the main crops grown, playing an important role in the tropical economy and rural development. According to data provided by the National Bureau of Statistics of China, the planting area for banana in Hainan was 3.4 × 10^4^ hectares in 2022, the annual banana production was around 1.15 × 10^6^ tonnes, and the output value was more than CNY 2.99 billion. ‘Pisang Mas’ (*Musa acuminata*, AA group) is recognized as a high-grade banana variety because of its small fruit, bright color, and unique taste; its average price is CNY 10–14 per kg, above that for a typical banana, CNY 4–6 per kg. The planting area in Hainan has reached 4.07 × 10^3^ hectares, accounting for 81.33% of the total planting area in China. However, as a perennial crop, vegetative propagation promotes the accumulation and adaptation of pathogens in banana plants, which seriously affects their growth and metabolism, thereby causing decreased yield and lower disease resistance [3]. The yield and quality of banana fruit are the most seriously affected by fungal diseases, as is currently being observed worldwide. Besides the difficulties posed by banana fusarium wilt, banana production also faces serious challenges due to foliage diseases like Sigatoka and freckle, which reduce the yield and increase the production costs [3].

In 2021–2022, a new leaf spot disease was observed in plants of the banana variety ‘Pisang Mas’ in three orchards (2.8 ha) in Hainan, China, which caused brown ovoid or lens-shaped lesions on their leaves and induced rapid leaf senescence; the causal agent was identified as *Bipolaris oryzae* (Breda de Haan) Shoemaker [4]. In the past three years, this disease has been prevalent in the main ‘Pisang Mas’ planting areas in Hainan, including the Chengmai, Lingao, Danzhou, and Ding’an counties. According to a field survey we conducted in Hainan, the severity of this disease has been increasing in areas growing this variety, and the rates of diseased plants and leaves in some ‘Pisang Mas’ orchards can reach 100% and 85%, respectively, in the rainy season, with estimated yield losses of 10–15%. The survey also found that *B. oryzae* caused slight harm to other banana varieties, such as ‘Cavendish’ (*Musa acuminata*, AAA group), ‘Pisang Awak’ (*Musa* spp., ABB group), and Dajiao (*Musa* spp., ABB group). There is still no banana variety resistant to foliage disease in China, and chemical control remains the main means of management. Furthermore, international research on the protective and curative control of foliage disease has mainly concentrated on Sigatoka leaf spot and freckle, while effective fungicides against other foliage diseases have rarely been discussed [3,5].

As *B.* *oryzae* is a new etiological agent causing severe leaf spot in the banana variety ‘Pisang Mas’ in Hainan, China, it is important to understand its biological characteristics and disease epidemiology, as well as the sensitivity of its isolates to the fungicides commonly used to control leaf spot disease in banana. The aim of this study was to assess this pathogen’s responses to important nutritional and environmental factors and its sensitivity to fungicides to understand its epidemic behavior in the field and provide a reference for the protective and curative control of this disease.

## 2. Materials and Methods

### 2.1. Pathogenic Fungal Strain, Inoculum Preparation, and Fungicides

The pathogenic fungus used in this study was *B.* *oryzae* FSBO2, obtained from the Hainan Provincial Key Laboratory of Pests Detection and Control for Tropical Agriculture, Environment and Plant Protection Institute, Chinese Academy of Tropical Agricultural Sciences, which was isolated from diseased banana plants in Hainan, China, in 2022 and subsequently identified as *Bipolaris oryzae* [4]. Strain FSBO2 was cultured on potato dextrose agar (PDA; 200 g/L of potato, 20 g/L of glucose, and 18 g/L of agar in distilled water) plates at 28 °C for 3 days in darkness to supply the inoculum.

Twelve fungicides, purchased from a local distributor, were selected for the preliminary screening of the *B.* *oryzae* pathogenic strain FSBO2 (Table 1) and diluted with sterile water in a series in preparation for later experiments. These fungicides were selected because they are currently registered for banana disease management in China.

### 2.2. The Effect of the Medium on the Mycelial Growth and Sporulation

We studied the mycelial growth and sporulation of *B. oryzae* on eight different media: PDA, potato sucrose agar (PSA; 200 g/L of potato, 20 g/L of sucrose, and 18 g/L of agar in distilled water), oatmeal agar (OA; 30 g/L of oatmeal and 18 g/L of agar in distilled water), carrot agar (CA; 200 g/L of carrot and 18 g/L of agar in distilled water), cornmeal agar (CMA; 30 g/L of cornmeal and 18 g/L of agar in distilled water), banana leaf extract agar (BLEA; 200 g/L of banana leaves and 18 g/L of agar in distilled water), malt extract agar (MEA; 30 g/L of malt extract and 18 g/L of agar in distilled water), and Czapek’s agar (CZA; 30 g/L of sucrose, 2 g/L of NaNO_3_, 1 g/L of K_2_HPO_4_, 0.5 g/L of MgSO_4_, 0.5 g/L of KCl, 0.01 g/L of FeSO_4_, and 18 g/L of agar in distilled water).

Mycelial plugs (5 mm in diameter) excised from the actively growing margins of 3-day-old *B. oryzae* FSBO2 colonies were placed upside down in the center of Petri dishes containing the different media and then incubated in darkness at 28 °C. After 3 days, the colony diameters were manually measured in two perpendicular directions. Three replicates per treatment were used, and the experiments were performed twice. After 12 days, the surfaces of the colonies were scraped using a sterile spatula, and the harvested fungal biomass was transferred to a 10 mL tube with 5 mL of sterile 0.1% (*v*/*v*) Tween 80. The suspension was shaken for 5 min with a laboratory vortex mixer to ensure the maximum detachment of the conidia from the mycelia and then filtered through double sterile gauze to remove the mycelia. The harvested conidia were washed twice and resuspended in sterile water. The conidia were counted using a hemacytometer under a microscope, and the conidium production was expressed as the number of conidia per milliliter (conidia/mL) of the colony, calculated using the formula for conidium production (conidia/mL) = X × N × 5 × 10^4^, where “X” is the dilution fold and “N” is the number of conidia in five squares of the hemacytometer [6].

### 2.3. The Effect of the Carbon and Nitrogen Sources on the Mycelial Growth and Sporulation

The mycelium growth and sporulation of *B. oryzae* on different carbon and nitrogen sources were determined in a CZA medium by replacing sucrose or NaNO_3_ with other carbon or nitrogen sources, respectively. CZA was used as the basal medium, in which the sucrose was replaced with equal mass concentrations of fructose, soluble starch, maltose, mannitol, lactose, sorbitol, and glucose to prepare media with different carbon sources. The NaNO_3_ in the basal medium was replaced with equal mass concentrations of beef extract, (NH_4_)_2_HPO_4_, NH_4_Cl, (NH_4_)_2_SO_4_, yeast extract, urea, and tryptone to prepare media with different nitrogen sources. Mycelial plugs (5 mm in diameter) excised from the actively growing margins of 3-day-old *B. oryzae* FSBO2 colonies were placed upside down in the center of CZA plates containing different carbon and nitrogen sources and then incubated at 28 °C in darkness. Using the same method as described above, the colony diameters and conidium production were determined after 3 and 12 days of incubation, respectively. Three replicates per treatment were used, and experiments were performed twice.

### 2.4. The Effect of Temperature on the Mycelial Growth and Sporulation

To evaluate the mycelial growth and sporulation of *B. oryzae* at different temperatures, mycelial plugs (5 mm in diameter) excised from the actively growing margins of 3-day-old colonies of *B. oryzae* FSBO2 were placed upside down at the center of the PDA plates and then incubated at 5 °C, 10 °C, 15 °C, 20 °C, 25 °C, 30 °C, 35 °C, and 40 °C in darkness. Using the same method as above, the colony diameters and conidium production were determined after 3 and 12 days of incubation, respectively. Three replicates per treatment were used, and the experiments were performed twice.

### 2.5. The Effect of pH on the Mycelial Growth and Sporulation

To clarify the mycelial growth and sporulation of *B. oryzae* at different pH levels, the pH value of the PDA was adjusted to 4, 5, 6, 7, 8, 9, and 10 with a 1 mol·L^−1^ H₃PO₄ solution or a 1 mol·L^−1^ NaOH solution. Mycelial plugs (5 mm in diameter) excised from the actively growing margins of 3-day-old colonies of *B. oryzae* FSBO2 were placed upside down at the center of the PDA plates at different pH values and then incubated at 28 °C in darkness. Using the same method as above, the colony diameters and conidium production were determined after 3 and 12 days of incubation, respectively. Three replicates per treatment were used, and the experiments were performed twice.

### 2.6. The Effect of Photoperiod on the Mycelial Growth and Sporulation

To assay the effects of photoperiod on *B. oryzae*, mycelial plugs (5 mm in diameter) excised from the actively growing margins of 3-day-old colonies of *B. oryzae* FSBO2 were placed upside down at the center of the PDA plates and then incubated at 28 °C under 5 different light treatments (24 h dark; 8 h light/16 h dark; 12 h light/12 h dark; 16 h light/8 h dark; 24 h light). Using the same method as above, the colony diameters and conidium production were determined after 3 and 12 days of incubation, respectively. Three replicates per treatment were used, and the experiments were performed twice.

### 2.7. Determination of the Mycelial Lethal Temperature

Mycelial plugs (0.5 cm in diameter) excised from the actively growing margins of 3-day-old *B. oryzae* FSBO2 colonies were transferred to a sterilized centrifuge tube containing 2 mL of sterile water. The tubes were placed in water baths preheated to different temperatures (25 °C, 40 °C, 45 °C, 50 °C, 55 °C, 60 °C, 65 °C, and 70 °C) for 10 min and then cooled to room temperature. The treated mycelial plugs were transferred to the center of the PDA plates for constant temperature cultivation at 28 °C in darkness, and their growth status was observed daily. Three replicates per treatment were used, and the experiments were performed twice. After the approximate lethal mycelial temperature was obtained, the temperature gradient interval was reduced to 1 °C to determine it more precisely.

### 2.8. Screening of Indoor Fungicides

The in vitro antifungal activity of 12 fungicides against *B.* *oryzae* was determined using the mycelial growth inhibition method [7]. PDA plates were prepared by mixing 98 mL of the medium (at ~55 °C) with 2 mL of each fungicide dilution at various concentrations, and we poured 10 mL of the mixed solution into sterilized Petri dishes. The final concentrations of each fungicide tested in this study are listed in Table 1. Mycelial plugs (0.5 mm in diameter) excised from the actively growing margins of 3-day-old *B. oryzae* FSBO2 colonies were placed upside down in the center of the PDA plates containing fungicides with different concentrations. Mycelial plugs transferred to PDA plates without fungicides were used as the control (CK). All the plates were incubated in the dark at 28 °C for 3 days, and the colony diameters were manually measured in two perpendicular directions. Three replicates per treatment were used, and the experiments were performed twice. The percentage of inhibition of mycelial growth (PIMG) was calculated for each combination of isolate-fungicide concentration using the following formula: PIMG (%) = [(mean colony diameter of the control − mean colony diameter of the treatment)/(mean colony diameter of the control − 5)] × 100 [8,9].

### 2.9. Control Efficacy of Fungicides

According to the results of the indoor toxicity test, 125 g/L of epoxiconazole SC, 500 g/L of iprodione SC, and 30% difenoconazole-azoxystrobin SC had better inhibitory effects and were selected for a control efficacy test. The highest recommended dosages of these fungicides were used as the treatment concentrations: 250 μg/mL for 125 g/L of epoxiconazole SC, 1667 μg/mL for 500 g/L of iprodione SC, and 250 μg/mL for 30% difenoconazole-azoxystrobin SC. The control efficacy (protective and curative) of fungicides against *B.* *oryzae* on banana leaves was assessed through pot experiments. The leaves of 7-leaf-old ‘Pisang Mas’ potted seedlings were rinsed under running tap water for 5 min, surface-disinfected in a 2% sodium hypochlorite solution for 30 sec, rinsed in sterile water three times, and then allowed to air-dry. The leaves on the selected potted seedlings were sprayed with three fungicide solutions at different treatment concentrations. Plants sprayed with sterile water served as controls. Each treatment was sprayed until runoff using a hand-held sprayer, and the plants were air-dried. The fungicides were applied 12 h after (curative treatment) or 12 h prior to inoculation (protective treatment).

Mycelial plugs (5 mm in diameter) excised from the actively growing margins of 3-day-old *B. oryzae* FSBO2 colonies were placed upside down on the leaves and covered with sterile, moist cotton. The inoculated leaves were wrapped individually with plastic bags to maintain high relative humidity, and the plants were kept at 26 °C in a greenhouse. The cotton and mycelial plugs were removed at 12 h post-inoculation, and the leaves were kept wrapped in plastic bags. The disease severity (DS) was then determined by measuring the lesion diameter in two perpendicular directions 7 days after inoculation. The experiment was performed twice, with six inoculation sites per leaf, two inoculated leaves on each plant, and three plants per treatment. The protective and curative control efficacies (CEs) were calculated using the following formula: CE (%) = [(mean lesion diameter of the control − mean lesion diameter with the treatment)/mean lesion diameter of the control] × 100 [8,10].

### 2.10. Statistical Analysis

The variance and means of all the data were calculated using Excel software, version 2021, and presented as the mean ± the SD (standard deviation of the mean). Virulence regression equations were established using the logarithm (log x) of the fungicide concentration as the independent variable and the probability value of the mycelial growth inhibition rate (y) as the dependent variable. The EC_50_ (effective concentration for 50% inhibition of mycelial growth) values of different fungicides were calculated using these equations using IBM SPSS Statistics software, version 27.0 (IBM Corp., Armonk, NY, USA). A one-way analysis of variance (ANOVA) and Duncan’s test (*p* < 0.05) were used to analyze the differences between the variables, including the EC_50_ values and different culture conditions, such as the culture medium, carbon and nitrogen sources, temperature, pH, and photoperiod. Graphical representations were created using Excel software, version 2021.

## 3. Results

### 3.1. Effects of the Media on the Mycelial Growth and Sporulation

**Figure 1:**
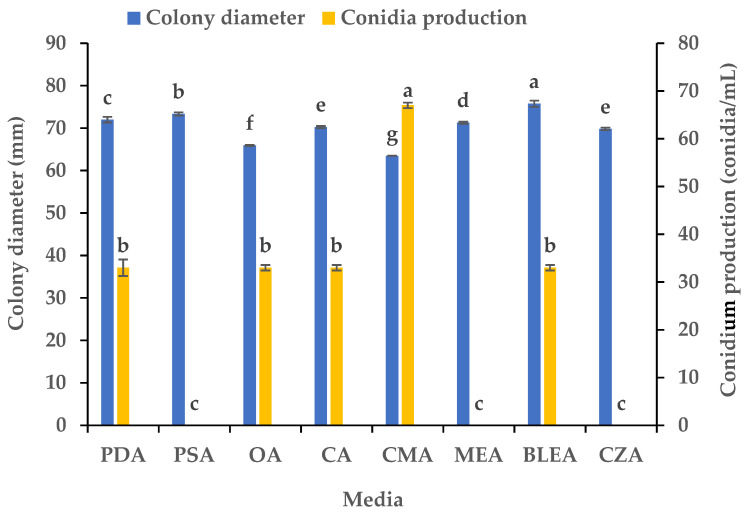
Effects of culture media on the mycelial growth and sporulation of *Bipolaris oryzae* FSBO2. PDA, potato dextrose agar; PSA, potato sucrose agar; OA, oatmeal agar; CA, carrot agar; CMA, cornmeal agar; MEA, malt extract agar; BLEA, banana leaf extract agar; CZA, Czapek’s agar. Different lowercase letters (a, b, c, d, e, f, g) indicate significant differences (*p* < 0.05) according to ANOVA. Data are presented as the mean ± SE (*n* = 3).

We studied the influence of eight media on the mycelial growth and sporulation of *B.* *oryzae* from banana plants affected by leaf spot. The results indicated that there were significant differences in the growth and sporulation of the *B.* *oryzae* strain FSBO2 on different media (Figure 1). The mycelium growth rate was the fastest on BLEA, with a significantly larger average colony diameter (75.75 mm) (*p* < 0.05) than that observed on the other media. The mycelial growth was also rapid on PSA and PDA, although the colony diameters on these media were slightly smaller than those on BLEA. The growth rate on MEA, CA, and OA was slower, resulting in significantly smaller colony diameters than those on the other media. The mycelial growth was the slowest on CMA (63.5 mm). In terms of sporulation, CMA provided the highest yield of *B.* *oryzae* spores and a conidial production of 67 conidia/mL, making it the best medium, followed by PDA, OA, CA, and BLEA. Notably, PSA, MEA, and CZA did not support any spore production at all (Figure 1).

### 3.2. Effects of Carbon and Nitrogen Sources on the Mycelial Growth and Sporulation

**Figure 2:**
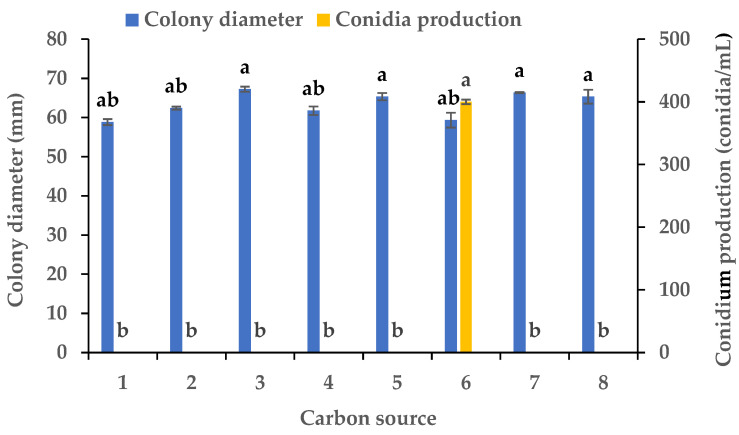
Effects of carbon sources on the mycelial growth and sporulation of *Bipolaris oryzae* FSBO2: 1, fructose; 2, soluble starch; 3, sucrose; 4, maltose; 5, mannitol; 6, lactose; 7, sorbitol; 8, glucose. Different lowercase letters (a, b) indicate significant differences (*p* < 0.05) according to ANOVA. Data are presented as the mean ± SE (*n* = 3).

**Figure 3:**
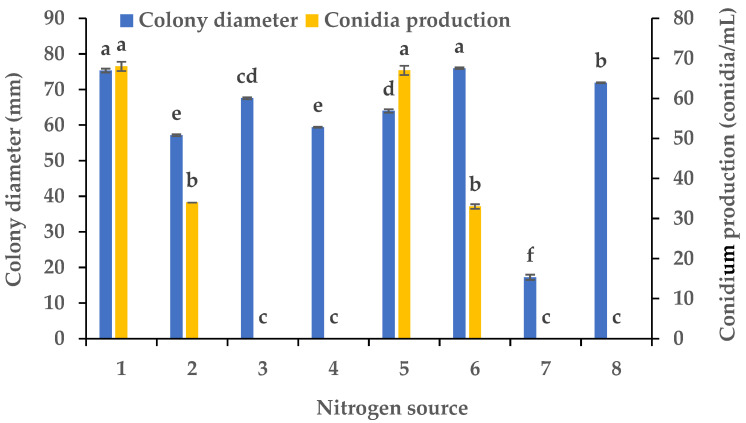
Effects of nitrogen sources on the mycelial growth and sporulation of *Bipolaris oryzae* FSBO2: 1, beef extract; 2, (NH_4_)_2_HPO_4_; 3, NaNO_3_; 4, NH_4_Cl; 5, (NH_4_)_2_SO_4_; 6, yeast extract; 7, urea; 8, tryptone. Different lowercase letters (a, b, c, d, e, f) indicate significant differences (*p* < 0.05) according to ANOVA. Data are presented as the mean ± SE (*n* = 3).

Experiments were conducted using CZA as a basal medium to investigate the influence of eight carbon and eight nitrogen sources on the mycelial growth and sporulation of *B.* *oryzae* from banana plants affected by leaf spot. Among the eight carbon sources tested, *B.* *oryzae* cultures in the medium containing sucrose, sorbitol, mannitol, and glucose exhibited the fastest hyphal extension rates, followed by those grown on the medium containing soluble starch and maltose (Figure 2). The colony growth rate was the lowest on the medium containing fructose, resulting in an average diameter of 58.83 mm. However, there was no significant difference in mycelial growth between the eight carbon sources tested. Among the eight nitrogen sources tested, *B.* *oryzae* cultures in the medium containing yeast and beef extracts exhibited the fastest hyphal extension rates, with average colony diameters on Day 3 of 76 mm and 75.3 mm, respectively, followed by those grown on the medium containing tryptone, which had a significantly larger colony diameter (*p* < 0.05) than the colonies on other nitrogen sources (Figure 3). The rate of utilization by FSBO2 was the lowest for urea, with an average colony diameter of only 17.3 mm. Only lactose supported the sporulation of *B.* *oryzae*, with a conidial production of 400 conidia/mL (Figure 2). Suitable nitrogen sources for the sporulation of *B.* *oryzae* were beef extract and (NH_4_)_2_SO_4_, with beef extract being optimal due to a conidial production of 68 conidia/mL resulting from its use, while no sporulation was recorded in the presence of the other four nitrogen sources (Figure 3).

### 3.3. Effects of Various Temperatures on the Mycelial Growth and Sporulation

**Figure 4:**
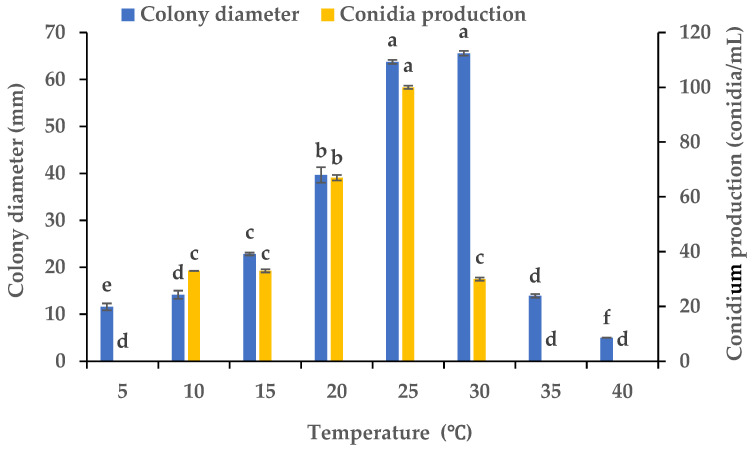
Effects of temperature on the mycelial growth and sporulation of *Bipolaris oryzae* FSBO2. Different lowercase letters (a, b, c, d, e, f) indicate significant differences (*p* < 0.05) according to ANOVA. Data are presented as the mean ± SE (*n* = 3).

Using PDA plates kept at varying temperatures, we evaluated the influence of the temperature on the mycelial growth and sporulation of *B.* *oryzae* from banana plants affected by leaf spot. The results indicated that the temperature had a significant effect on the mycelial growth and sporulation of *B.* *oryzae* FSBO2. The ideal temperatures for mycelial growth were in the range of 25 °C to 30 °C, and the optimal temperature was 30 °C, at which the mycelial growth significantly surpassed that under other temperature treatments (*p* < 0.05) (Figure 4). The mycelial growth was notably slower at 5 °C, 10 °C, 15 °C, and 35 °C and completely absent at 40 °C. The ideal temperatures for sporulation were between 20 °C and 25 °C, and the optimal temperature was 25 °C, at which the sporulation significantly exceeded that under other temperature treatments (Figure 4). Sporulation was completely absent at 5 °C, 35 °C, and 40 °C.

### 3.4. Effects of Various pH Values on the Mycelial Growth and Sporulation

**Figure 5:**
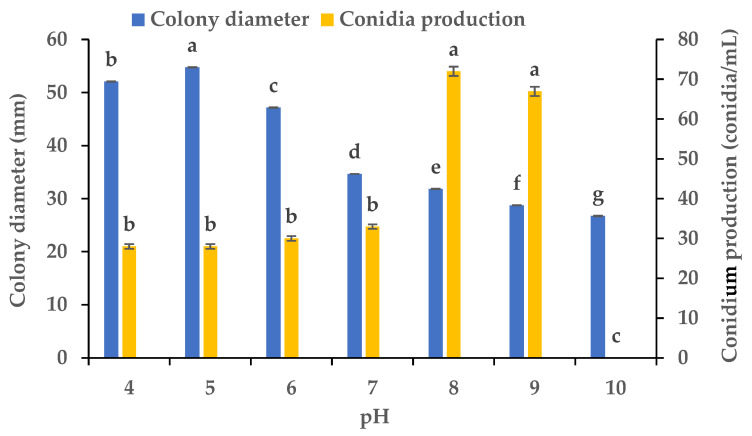
Effects of pH on the mycelial growth and sporulation of *Bipolaris oryzae* FSBO2. Different lowercase letters (a, b, c, d, e, f, g) indicate significant differences (*p* < 0.05) according to ANOVA. Data are presented as the mean ± SE (*n* = 3).

Using PDA plates with varying pH levels, we clarified the influence of the pH on the mycelial growth and sporulation of *B.* *oryzae* from banana plants affected by leaf spot. The results showed that there were significant differences in the colony diameter and spore production of *B.* *oryzae* under different pH levels (Figure 5). The FSBO2 strain could grow and produce spores at pH values from 4 to 9. The ideal pH values for mycelial growth were 4 to 6, among which the optimal pH was 5, with the growth at this pH significantly (*p* < 0.05) surpassing that under other pH treatments (Figure 5). The mycelial growth was notably slower at pH 7, 8, 9, and 10. The ideal pH values for sporulation were 8 and 9, with the sporulation at these values significantly (*p* < 0.05) surpassing that under other pH treatments, and the optimal pH was 8 (Figure 5). The sporulation was notably reduced at pH 4, 5, 6, and 7 and completely absent at pH 10.

### 3.5. Effects of Various Photoperiods on the Mycelial Growth and Sporulation

**Figure 6:**
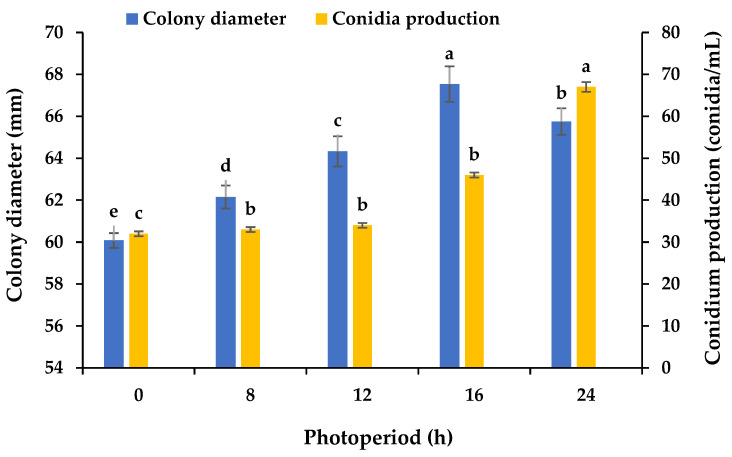
Effects of photoperiod on the mycelial growth and sporulation of *Bipolaris oryzae* FSBO2. 0 h, 24 h dark; 8 h, 8 h light/16 h dark; 12 h, 12 h light/12 h dark; 16 h, 16 h light/8 h dark; 24 h, 24 h light. Different lowercase letters (a, b, c, d, e) indicate significant differences (*p* < 0.05) according to ANOVA. Data are presented as the mean ± SE (*n* = 3).

Using PDA plates and varying light periods, we assayed the influence of light on the mycelial growth and sporulation of *B.* *oryzae* from banana plants affected by leaf spot. The results showed that 16 h light/8 h dark was the most favorable for mycelial growth, with significant differences (*p* < 0.05) between the growth with this photoperiod and the other tested photoperiods (Figure 6), followed by 24 h light. The mycelial growth was slower under the other light treatments. A photoperiod of 24 h light was found to be favorable for the sporulation (67 conidia/mL) of *B.* *oryzae*, with significant differences (*p* < 0.05) between the sporulation with this photoperiod and the other tested photoperiods (Figure 6). The sporulation was reduced under the other light treatments (Figure 6).

### 3.6. Determination of the Lethal Temperature for Mycelia

**Figure 7:**
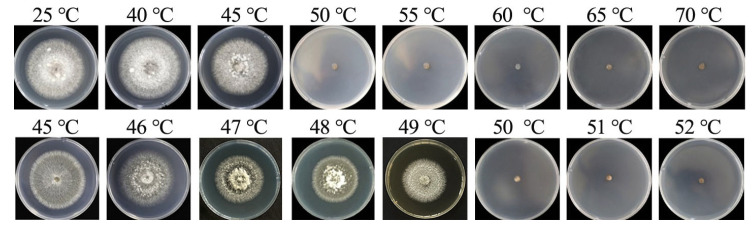
Lethal temperatures for mycelia of *B.* *oryzae*.

Through incubation in water baths at varying temperatures, we determined the mycelial lethal temperature for *B.* *oryzae* from banana plants affected by leaf spot. The results indicated that mycelial plugs could grow and form colonies when soaked in water at temperatures ranging from 25 °C to 45 °C for 10 min (Figure 7). They did not grow when exposed to temperatures of 50 °C and above. To further refine the precision of the temperature threshold, the gradient was narrowed to 1 °C between 45 °C and 52 °C and the tests were repeated, revealing that the fungal mycelial plugs could not grow normally after being soaked in water at between 50 °C and 52 °C for 10 min (Figure 7); this indicated that the lethal temperature for the fungus was 50 °C with a 10 min exposure.

### 3.7. Antifungal Activities of Fungicides

**Figure 8:**
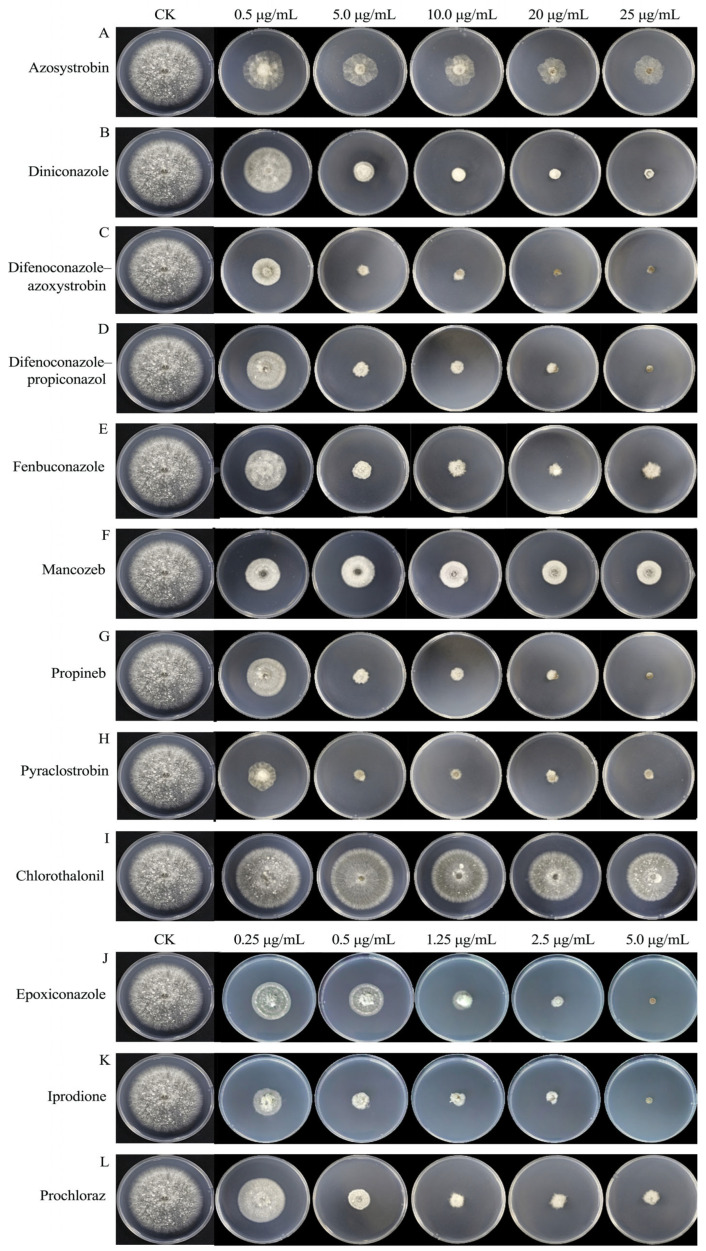
Effects of 12 fungicides on the mycelial growth of *B.* *oryzae* FSBO2. (**A**–**I**) Final concentration of nine fungicides (0.5 μg/mL, 5.0 μg/mL,10.0 μg/mL, 20 μg/mL, and 25 μg/mL). (**J**–**L**) Final concentration of three fungicides (0.25 μg/mL, 0.5 μg/mL, 1.25 μg/mL, 2.5 μg/mL, and 5.0 μg/mL). CK represents PDA plates without fungicides.

We assayed the antifungal activities of 12 fungicides commonly used in agricultural fields against *B.* *oryzae* using the growth rate method. The results showed that all 12 fungicides exhibited certain inhibitory effects on *B.* *oryzae*, which increased with rising fungicide concentrations, and there were significant differences in the inhibitory effects between the tested fungicides (Table 1, Figure 8).

The correlation coefficients from the toxicity regression equations for the 12 fungicides ranged from 0.9 to 1.0, indicating a strong correlation between the dosage of the fungicides and their inhibitory effects (Table 2). The representative isolate *B.* *oryzae* FSBO2 showed differential sensitivity to the 12 fungicides tested (Table 2). Among the 12 fungicides, 500 g/L of iprodione SC had the highest inhibition rate, with an EC_50_ value of 0.08 μg/mL. This was followed by 30% difenoconazole-azoxystrobin SC, 125 g/L of epoxiconazole SC, 25% pyraclostrobin SC, 450 g/L of prochloraz EW, 24% fenbuconazole SC, and 30% difenoconazole-propiconazol EC, which also showed strong inhibitory effects against *B.* *oryzae*, with EC_50_ values ranging from 0.13 μg/mL to 0.72 μg/mL. Moderate effects were observed with 12.5% diniconazole WP, 250 g/L of azoxystrobin SC, 70% propineb WP, and 43% mancozeb SC, which resulted in EC_50_ values ranging from 1.19 μg/mL to 5.21 μg/mL. The lowest inhibition rates were found with 40% chlorothalonil SC, with an EC_50_ value of 155.98 μg/mL. Moreover, using the regression equation, we found that the slope of 500 g/L of iprodione SC was the largest at 5.8348 μg/mL and that of 40% chlorothalonil SC was the smallest at 3.5974 μg/mL. This indicated that *B.* *oryzae* was the most sensitive to 500 g/L of iprodione SC and the least sensitive to 40% chlorothalonil SC.

### 3.8. Protective and Curative Activity of Fungicides

In the artificial inoculation pot experiment, 125 g/L of epoxiconazole SC, 500 g/L of iprodione SC, and 30% difenoconazole-azoxystrobin SC at the highest recommended dosages of 250 μg/mL, 1667 μg/mL, and 250 μg/mL, respectively, exhibited an excellent protective control efficacy of 100% against *B.* *oryzae* with application 12 h before inoculation (Table 3). There was no significant difference (*p* > 0.05) in the protective efficacy among these three fungicides.

Regarding the fungicides’ curative activity, 125 g/L of epoxiconazole SC at 250 μg/mL and 500 g/L of iprodione SC at 1667 μg/mL showed moderate efficacies of 64% and 60%, respectively, when applied 12 h after inoculation, which were significantly better than (*p* < 0.05) the 36% efficacy of 30% difenoconazole-azoxystrobin SC (Table 3).

## 4. Discussion

*Bipolaris oryzae* is a multi-host pathogen which causes severe leaf spot in more than 26 important plant species from 40 countries, including graminaceous (e.g., rice, *Leersia hexandra*, and *Zizania latifolia*) and nongraminaceous (e.g., peanut, switchgrass, and *Aechmea tayoensis*) crops and some trees (e.g., Chinese fir and *Cocos nucifera*) [11,12,13,14,15,16,17]. Cases of rice brown spot caused by *B.* *oryzae* occur worldwide and are known to cause substantial quantitative and qualitative grain yield losses [18]. Besides leaf spot, this pathogen is also related to severe seed rot in rice and shoot blight in Chinese fir [16,19]. The author’s team was the first to report the occurrence of leaf spot caused by *B.* *oryzae* in the banana variety ‘Pisang Mas’ [4].

There are significant differences in the biological characteristics of *B.* *oryzae* isolates from different host plants and geographical areas [20,21,22], and various nutritional and environmental factors have been observed to influence the pathogen’s survival and disease development [18,23,24,25]. Moreover, a number of fungicides have been evaluated against *B.* *oryzae* under in vitro and in vivo conditions [22,26,27,28,29], revealing that the protective and control effects of various fungicides on crops in different geographic regions are inconsistent, which may be related to this pathogen having varying sensitivities to chemical fungicides.

This pathogen’s nutrition and environment seemed to play an important role in the regulation of its mycelial growth and sporulation in the banana variety ‘Pisang Mas’ in China (Figure 1, Figure 2, Figure 3, Figure 4, Figure 5 and Figure 6). The fungus grew normally on the tested media and could produce spores on five of the media but not on potato sucrose agar, malt extract agar, and Czapek’s agar. The most suitable medium for promoting mycelial growth was banana leaf extract agar, while cornmeal agar was the most suitable for promoting sporulation out of the tested media (Figure 1). These results are similar to those obtained for a *B.* *oryzae* isolate from rice, for which the optimal medium promoting mycelial growth was paddy leaf extract agar [21], but are different from those for an isolate causing brown spot in *Zizania latifolia*, for which the optimal medium for promoting sporulation was water bamboo culm juice agar [30]. The fungus grew vigorously on the Czapek’s agar medium with the eight carbon sources tested, while lactose was the only effective source promoting sporulation (Figure 2). The *B.* *oryzae* isolated in this study grew vigorously on the Czapek’s agar medium with seven of the tested nitrogen sources but not urea, while beef and yeast extracts and nitrate were effective in promoting sporulation (Figure 3). The fungus could adapt to a wide range of temperatures and was capable of growing within a temperature range of 5 °C to 35 °C, and the ideal temperature range for mycelial growth was 25 °C to 30 °C. For sporulation, the ideal temperature range was 10 °C to 30 °C, with an optimal temperature of 25 °C (Figure 4). The average monthly temperature from March to November is 20 °C to 30 °C in the banana-growing regions in Hainan Province, coinciding with the two banana foliage disease epidemic seasons in the field [31,32]. In the PDA medium, the fungus was capable of growing and producing spores within pH ranges of 4–10 and 4–9, respectively (Figure 5), showing strong adaptability to a variety of pH values. Excessive alkalinity was not conducive to mycelial growth and sporulation, which was consistent with the results obtained on the mycelial growth of the pathogen causing leaf spot in cane shoots [22]. The fungus could grow normally under five different photoperiods, among which the optimal conditions for mycelial growth and sporulation were 16 h light/8 h dark and 24 h light, respectively (Figure 6), aligning with the local photoperiod conditions in Hainan Province, China. This indicates that the strain of *B.* *oryzae* isolated in this study requires high light levels, which is consistent with the results obtained for *B.* *oryzae* from *Leersia hexandra* [33]. On the basis of submersion in a water bath for 10 min, the lethal temperature of the pathogen was 50 °C (Figure 7).

Bioassays were conducted to evaluate the inhibitory effects of 12 fungicides on the growth of *B.* *oryzae*, the causal agent of leaf spot in banana. The results demonstrated that all 12 fungicides exhibited inhibitory effects on the mycelial growth of *B.* *oryzae*, with the degree of inhibition increasing with the concentration of the fungicides (Table 1, Figure 8). The toxicity of these agents was significantly different. The toxicity of 500 g/L of iprodione SC to *B. oryzae* was the highest, with an EC_50_ of 0.08 μg/mL (Figure 8K, Table 2). This indicates that diformimide fungicides have a strong inhibitory effect on *B.* *oryzae*, consistent with results obtained for leaf spot caused by *B*. *zeae* in *Axonopus compressus* [34]. Six fungicides—30% difenoconazole-azoxystrobin SC, 125 g/L of epoxiconazole SC, 25% pyraclostrobin SC, 450 g/L of prochloraz EW, 24% fenbuconazole SC, and 30% difenoconazole-propiconazol EC—showed very high toxicity against *B.* *oryzae*, with EC_50_ values of 0.13-0.72 μg/mL (Figure 8, Table 2). Meanwhile, 12.5% diniconazole WP, 250 g/L of azoxsystrobin SC, 70% propineb WP, and 43% mancozeb SC also showed high toxicity to *B.* *oryzae*, with EC_50_ values of 1.19-5.21 μg/mL. These findings are consistent with results on the sensitivity to fungicides of *B. oryzae* isolated from cane shoot leaf spot and rice brown spot [22,25]. Compared with other fungicides, 40% chlorothalonil SC exhibited the lowest toxicity, with an EC_50_ value of 155.98 μg/mL (Figure 8I, Table 2); therefore, it should not be used as a control agent. The artificial inoculation pot experiment showed that 125 g/L of epoxiconazole SC, 500 g/L of iprodione SC, and 30% difenoconazole-azoxystrobin SC had the best protective effects against banana leaf spot caused by *B. oryzae*, and the protective control efficacy was 100% when the dosages were 250 μg/mL, 1667 μg/mL, and 250 μg/mL, respectively. The curative effects of 125 g/L of epoxiconazole SC and 500 g/L of iprodione SC for banana leaf spot caused by *B. oryzae* were the best, and the curative control efficacy was more 60% when the dosages were 250 μg/mL and 1000 μg/mL, respectively. Given that the results of laboratory bioassays and inoculation pot experiments are not always consistent with the control efficacy in the field, further trials must be conducted under field conditions.

## 5. Conclusions

This study clarified the biological characteristics of *B.* *oryzae*, the pathogen causing leaf spot in the banana variety ‘Pisang Mas’ in Hainan Province, and found that different nutritional and environmental conditions can affect the growth and sporulation of *B.* *oryzae* from banana plants affected by leaf spot. The indoor toxicity analysis showed that 125 g/L of epoxiconazole SC, 500 g/L of iprodione SC, and 30% difenoconazole-azoxystrobin SC exhibited strong antifungal activity against *B.* *oryzae*. Additionally, they showed the best protective effects and could be used as protective agents against banana leaf spot caused by *B.* *oryzae*. The curative control efficacies of 125 g/L of epoxiconazole SC and 500 g/L of iprodione SC were better than that of 30% difenoconazole-azoxystrobin SC. This study provides a reference for the comprehensive control of the banana leaf spot disease caused by *B.* *oryzae* in Hainan Province, China.

## Figures and Tables

**Table 1:** Names, manufacturers, experimental concentration gradients of twelve fungicides and their inhibition rates on *B.* *oryzae*.

Fungicide	Manufacturer	Concentration (μg/mL)	Inhibitory Rates (%)
250 g/L of azosystrobin SC	Syngenta Nantong Crop Protection Co., Ltd. (Nantong, China)	0.5	42.35
		5	56.34
		10	59.4
		20	62.61
		25	64.3
		CK	—
12.5% diniconazole WP	ADAMA Huifeng (Jiangsu) Co., Ltd. (Yancheng, China)	0.5	35.29
		5	72.03
		10	84.58
		20	89.94
		25	91.44
		CK	—
30% difenoconazole-azoxystrobin SC	Sipcam Agro USA (Shanghai, China)	0.5	63.14
		5	82.75
		10	88.63
		20	89.61
		25	91.58
		CK	—
30% difenoconazole-propiconazol EC	Sichuan Runer Technology Co., Ltd. (Jianyang, China)	0.5	46.27
		5	77.26
		10	81.18
		20	89.80
		25	94.38
		CK	—
24% fenbuconazole SC	Kedihua Agricultural Technology Co., Ltd. (Beijing, China)	0.5	46.54
		5	68.07
		10	74.51
		20	78.56
		25	80.47
		CK	—
43% mancozeb SC	Henan Xinnong Chemical Co., Ltd. (Zhengzhou, China)	0.5	6.40
		5	50.76
		10	71.80
		20	69.51
		25	88.11
		CK	—
70% propineb WP	Bayer Crop Science (China) Co., Ltd. (Beijing, China)	0.5	8.24
		5	49.15
		10	85.23
		20	89.54
		25	92.83
		CK	—
25% pyraclostrobin SC	Nanjing Huazhou Pharmaceutical Co., Ltd. (Nanjing, China)	0.5	58.43
		5	81.70
		10	83.92
		20	88.47
		25	89.15
		CK	—
40% chlorothalonil SC	SDS Biotech K. K. (Shanghai, China)	0.5	6.54
		5	11.63
		10	23.92
		20	28.89
		25	32.03
		CK	—
125 g/L of epoxiconazole SC	Shandong Jophne Biotechnology Co., Ltd. (Jinan, China)	0.25	45.23
		0.5	54.64
		1.25	70.59
		2.5	83.53
		5	89.41
		CK	—
500 g/L of iprodione SC	Guangdong Zhuoyue Biotechnology Co., Ltd. (Nanxiong, China)	0.25	61.31
		0.5	76.34
		1.25	82.61
		2.5	86.08
		5	100
		CK	—
450 g/L of prochloraz EW	Zhejiang Tianfeng Bioscience Co., Ltd. (Jinhua, China)	0.25	38.43
		0.5	73.86
		1.25	75.1
		2.5	78.04
		5	83.06
		CK	—

CK, PDA plates without fungicides.

**Table 2:** The inhibition effect of 12 fungicides on *B.* *oryzae*.

Fungicide	Regression Equation	Correlation Coefficient	EC_50_ (μg/mL)	95% Confidence Intervals (μg/mL)
250 g/L of azoxystrobin SC	y = 0.3247 x + 4.9126	0.9984	1.86	0.00–850.65
12.5% diniconazole WP	y = 1.0399 x + 4.9222	0.9981	1.19	0.09–14.88
30% difenoconazole-azoxystrobin SC	y = 0.6054 x + 5.5285	0.9937	0.13	0.00–387.124
30% difenoconazole-propiconazol EC	y = 0.9122 x + 5.1318	0.9802	0.72	0.02–21.01
24% fenbuconazole SC	y = 0.5551 x + 5.0835	0.9994	0.71	0.00–125.18
43% mancozeb SC	y = 1.4597 x + 3.9541	0.9785	5.21	1.62–16.78
70% propineb WP	y = 1.7118 x + 4.0682	0.9859	3.50	1.05–11.73
25% pyraclostrobin SC	y = 0.6009 x + 5.4159	0.9954	0.20	0.00–251.10
40% chlorothalonil SC	y = 0.6396 x + 3.5974	0.9549	155.98	0.11–221,352.16
125 g/L of epoxiconazole SC	y = 1.0358 x + 5.7245	0.9475	0.20	0.02–2.22
500 g/L of iprodione SC	y = 0.7636 x + 5.8348	0.9658	0.08	0.00–2.31
450 g/L of prochloraz EW	y = 0.6961 x + 5.1897	0.9753	0.53	0.02–20.90

**Table 3:** Protective and curative effects of three fungicides in pot experiments.

Treatment	Protective Activity		Curative Activity	
	Mean Lesion Diameter (mm)	Control Efficacy (%)	Mean Lesion Diameter (mm)	Control Efficacy (%)
125 g/L of epoxiconazole SC (250 μg/mL)	0 ± 0 b	100 ± 0 a	2.25 ± 0.56 b	64 ± 5.09 a
500 g/L of iprodione SC (1667 μg/mL)	0 ± 0 b	100 ± 0 a	2.5 ± 0.77 c	60 ± 4.86 a
30% difenoconazole-azoxystrobin SC (250 μg/mL)	0 ± 0 b	100 ± 0 a	4 ± 1.14 c	36 ± 2.57 b
CK	9.25 ± 3.3 a	–	6.25 ± 1.54 a	–

Different lowercase letters (a, b, c) in same column indicate significant differences (*p* < 0.05) according to ANOVA. Data are presented as the mean ± SE (*n* = 3).

## Data Availability

The data presented in this study are available within the article.
